# Contemporary Challenges of Nursing CPD: Time to change the model to meet citizens’ needs

**DOI:** 10.1002/nop2.941

**Published:** 2021-05-24

**Authors:** Carolyn Jackson, Kim Manley

**Affiliations:** ^1^ Director ImpACT Research Group Associate Professor Practice Transformation Faculty of Medicine and Health Sciences University of East Anglia Norwich UK; ^2^ CBE Professor of Practice Development & Co‐Director ImpACT Research Group Faculty of Medicine and Health Sciences University of East Anglia Norwich UK; ^3^ Emeritus Professor in the Faculty of Medicine, Health and Social Care Canterbury Christ Church University Canterbury UK

**Keywords:** citizens panels, co‐production, CPD impact, nursing CPD, transformation, workplace learning

## Abstract

The purpose of this paper is to present the evidence shared with a citizen Consensus panel detailing key issues associated with how nursing CPD can best influence the quality of health and social care experienced by citizens and communities. It presents a summary of contemporary theory, research and evidence of the effectiveness of nursing CPD and outlines four key challenges: (i) how to strengthen the focus on patient experience as the starting point for CPD; (ii) the lack of evidence of CPD effectiveness and accountability in its transfer to practice; (iii) evaluation of CPD effectiveness; and (iv) involving citizens in targeting CPD where it is most needed. It briefly describes the methods used to facilitate public consultation through a citizen Consensus panel as part of a collaborative project with the RCN Strategic Research Alliance in 2020 and outlines 7 themes identified as important by the public for future development. The main challenge for nursing is capitalizing on the workplace as a learning resource that can integrate learning with development, improvement, knowledge translation, inquiry and innovation. This requires skilled facilitators, particularly at meso‐ levels, and systems leaders with the full skillset to develop system‐wide cultures of learning that enable everyone to flourish and create good places to work. The paper concludes that the development of CPD process measures would indicate how CPD investment contributes to person‐centred, safe and effective care and system transformation and enable commissioners and education providers to optimize CPD’s full potential.

## 1 INTRODUCTION

Continuing professional development (CPD) for nursing is essential for career progression and maintaining person‐centred, safe and effective evidence‐informed care in the workplace (Jackson et al., 2015; Manley et al., 2018), yet is fraught with issues. It plays a crucial role in continued fitness to practice and patient safety, and maintaining professional standards, and is effective only to the extent that learning is transferred to the workplace (Marvin et al., 2010). In the United Kingdom (UK), nurses are required to undertake 12 hr of CPD annually, compared to other countries which average 30 hr (European Union Health Programme, 2013; Tran et al., 2014). Every three years the regulator requires revalidation, to demonstrate practice is up to date for public protection.

Despite the plethora of clinical and academic CPD programmes that lay claim to prepare the workforce to lead and facilitate improvements in patient safety and quality of care, most CPD focuses on individuals or teams, and there is little research evidence of; (i) what difference these programmes make in practice at the macro (system)‐, meso (service)‐ and micro (team)‐levels of the system,^1^ (ii) whether there is cost benefit for system investment and (iii) what impact these programmes have on outcomes such as career progression, staff well‐being, retention and sustainable innovation, or improvement in patient experience and outcomes. We argue in this paper that a holistic approach to workforce development at all levels of the system is required for quality care and transformation of services involving all interdependent partners, working with the complexity of practice contexts, using the workplace as the main resource for learning, development and improvement (Manley & Jackson, 2020; Manley et al., 2016, 2019; Martin & Manley, 2018). Four key issues associated with funding and commissioning future CPD inform recommendations about how nursing CPD budgets could be used more effectively, and how workforce development and transformation should be influenced by what matters to citizens.

## 2 THE EVIDENCE: CPD, ITS PURPOSE, INDICATORS AND OUTCOMES

### 2.1 Definition

There is no universally agreed definition of CPD, Box 1 provides a comprehensive summary for a term often used synonymously with continuing nursing education, lifelong learning and professional skills development (RCN, 2016).

BOX 1 **European Definition of CPD**

“The systematic maintenance, improvement and continuous acquisition and/or reinforcement of the life‐long knowledge, skills and competences of health professionals. It is pivotal to meeting patient, health service delivery and individual professional learning needs. The term acknowledges not only the wide‐ranging competences needed to practise high quality care delivery but also the multi‐disciplinary context of patient care” (EAHC, EU report 2013:6).

Traditional approaches to both formal and informal CPD undertaken in a wide range of settings, from the workplace, to the classroom, is content focused and places value on mandatory training and knowledge acquisition. It relies on the individual practitioner to use, implement and blend different types of evidence in the workplace (Jackson et al., 2015). Contextual factors (culture, evaluation and leadership) and holistic facilitation influence implementation (Graham et al., 2006; Kitson et al., 1998), with organizational learning, involving co‐production, a recent development (Rowley et al., 2012). In contrast, CPD that maximizes the opportunity to learn at work, through work and for work (Tynjala, 2013), using the workplace as the main resource for learning, development, innovation and improvement, helps to shape practice in real time and enables practitioners to make a meaningful contribution to their team, service and organization (Manley & Jackson, 2020; Manley et al., 2019).

### 2.2 Contemporary Research Evidence of CPD Impact

A recent rapid review of 39 international CPD studies (King et al., 2021), identified the factors that optimize CPD impact in nursing; including self‐motivation of learners, relevance to practice, preference for workplace learning, strong enabling leadership and a positive workplace culture. The findings indicated the interdependence of these factors when optimizing CPD impact on person‐centred care and outcomes.

Current challenges for nursing CPD include:

1. significant reductions in funding threaten the ability of nurses to meet the requirements for revalidation (NMC, 2017; RCN, 2018);
2. potential to be underprepared to supervise future nursing students in attaining the new NMC standards of proficiency, which identify the extended knowledge and skills required for registration (Council of Deans of Health, 2016; NMC, 2018; RCN, 2018);
3. impact of CPD budget on nursing workforce recruitment and retention (House of Commons Health Committee, 2018);
4. Recognized link between level of nursing qualification and patient safety with little attention on how access to CPD impacts safe and effective care (Aiken et al., 2018; European Union Health Programme, 2013);
5. Inability to access CPD influences patient safety and quality of care, which adversely affects job satisfaction, recruitment and retention (Aiken et al., 2018; Coventry, 2015).

Two key studies, summarize the international evidence of CPD impact across healthcare professions, repositioning the purpose of CPD as using the workplace as the main resource for learning, development and improvement (Illing et al., 2018; Jackson et al., 2015). Both use realist methodologies to identify the strategies that work and shed light on key issues which warrant further study.

The first study by Illing et al., (2018) aimed to identify how education and training of health and social care staff can transfer to practice and benefit patients based on evidence from 368 studies. Findings were tested with five NHS case studies, and a survey of 600 health and social care staff who stated how their education or CPD had benefitted patients. Illing et al., (2018) review showed a top‐down approach; illustrating how organizations can commission education or training that transfers to practice and benefits patients. However, the case studies and survey findings identified that the model could also be effective using a bottom‐up approach, showing how individuals and teams could focus on a patient problem and find the evidence to benefit patients (persuading organizations to provide the resources needed). Rhydderch et al., (2004) also recognized the process for change in the United Kingdom was primarily “top‐down”, and that greater encouragement should be given to ownership of change at the “lower” individual, team and organizational levels.

Illing et al., (2018) advocated a focus on developing teams rather than individuals in order to improve knowledge transfer and real‐time changes in the quality of care because of its impact on culture. This was evidenced by the observation that when new staff were exposed to existing staff behaviours, which had become embedded into regular practice following CPD, they adopted the same behaviours without needing the educational intervention.

Their four‐step model identifies how to facilitate staff learning and development in the workplace:

1. Training is designed to benefit patients and aligned to the strategic goals of the organization.
2. The learner is motivated, recognizing the importance and relevance of the training.
3. The learner successfully learns and has a desire to put the learning into practice.
4. Knowledge is transferred to practice, through a momentum for change. Training whole teams reduces resistance to change (Illing et al., 2018).

This model was intended to ensure formal education benefits patients, considering the importance of the workplace environment. It does however continue to focus on the concept of CPD as “training” as opposed to thinking about “learning” and its potential to transform the workforce. The study does not explore how patients and citizens can shape the CPD focus of the organization to address gaps in service provision, workforce development or quality of care.

The second study by Jackson et al., (2015) aimed to develop a multi‐professional framework to measure the impact of CPD, to evaluate whether learning has been effective in improving the quality of patient care and experience in the workplace, and in supporting sustainable practice transformation for all health and social care professions. The framework for effective CPD captured indicators at micro‐, meso‐ and macro‐levels across the system. Taking a whole systems approach to interprofessional learning, four new transformational theories (Table 1) were developed to describe and explain the relationships between what works (context = C), why it works (mechanisms = M) to achieve specific outcomes (O) of CPD learning in practice, linked to impact and potential indicators of effectiveness. Four CPD purposes were identified as:

1. enhancing individual professional practice essential for growing and retaining staff;
2. developing skills and knowledge to meet changing healthcare needs of the population (context);
3. getting best practice and evidence implemented to improve the standards of patient care and citizen well‐being;
4. transforming the workplace culture to enable implementation of shared values and learning in the workplace so that care is person‐centred, safe and effective (overarching purpose).

**TABLE 1:** CPD transformation theories (Jackson et al., 2015)

CPD theories	
Theory 1: Transformation of an Individual’s Professional Practice Through CPD	CPD that is work based within a context that is enabling, inquiring and supportive and learner‐driven, and centred on the provision of facilitated support and reflection and includes self‐assessment and a focus on self‐awareness will increase self‐confidence, self‐awareness, self‐efficacy, role clarity, and create a positive attitude to change with opportunities for role and career development.
Theory 2: Transformation of Skills to Meet Society’s Changing Healthcare Needs Through CPD	CPD that focuses on the transformation of skills to meet society’s changing healthcare needs embracing team and system assessment to identify gaps and expand skills to meet a changing healthcare context will be reflected in better service user experiences of continuity and consistency of service provision, better employability and opportunities for career progression for individuals, more effective teams better organizational/systems outcomes around integration, partnerships and more effective use of human resources.
Theory 3: Transformation of Knowledge Enabling Knowledge Translation Through CPD	CPD in workplace contexts that both support and encourage engagement with and use of different types of knowledge in everyday practice and active sharing through CPD strategies that focus on: using and blending multiple knowledges^a^ to inform professional decision‐making; skills in facilitating dialogue, active enquiry and evaluation; and, developing practical and theoretical knowledge fostering leadership, evaluation and culture will achieve knowledge rich cultures recognized by knowledge use and development, active inquiry, innovation and creativity.
Theory 4: Transformation of Workplace Culture/Context to Implement Workplace and Organizational Values and Purpose Relating to Person‐Centred, Safe and Effective Care Through CPD	CPD that takes place within contexts where there are shared values and purposes and organizational readiness that draws on CPD strategies which focus on: developing and implementing shared values; evaluating the experiences of service users and staff in relation to these values; and, developing skills in developing effective workplace cultures through leadership will achieve improved service user and provider experiences, outcomes and impact, sustained person‐centred, safe and effective workplace cultures and team effectiveness, increased employee commitment, organizational leadership and effectiveness.

^a^ Knowledges encompasses theoretical and practical knowledge, knowledge of the person being cared for/worked with, experience, expertise, artistry, creativity and local knowledge.

Workplace culture is critical to a learning organization and effective high performing teams as it impacts on whether staff thrive and flourish and quality services are provided (Cardiff et al., 2020; Jackson et al., 2015; Manley & Jackson, 2020; Manley et al., 2018).

## 3 CPD TRANSFORMATION THEORIES

The study concluded that both the workplace and organization impact on achieving CPD outcomes through the content focused on in terms of learning and development; and whether the workplace is valued and used as a resource for learning, development, innovation and improvement (Jackson et al., 2015). This is because there is often dissonance between what the organization or system aims to achieve and what front‐line teams are achieving, hence the need to focus on how interdependent service providers (meso‐level) enable front‐line teams to be effective.

The COVID‐19 pandemic has highlighted the challenges associated with rapid change in times of crisis and the impact on front‐line staff well‐being,^2^ services, organizations and systems delivering health and social care globally (Chaudry & Raza, 2020; Greenberg et al., 2020; Jackson et al., 2020, 2021; Maben & Bridges, 2020). Staff well‐being is an indicator of a flourishing workplace culture that impacts on staff commitment, resilience and retention, and in health care, it results in an experience of quality, person‐centred care (Maben et al., 2012).

**FIGURE 1:**
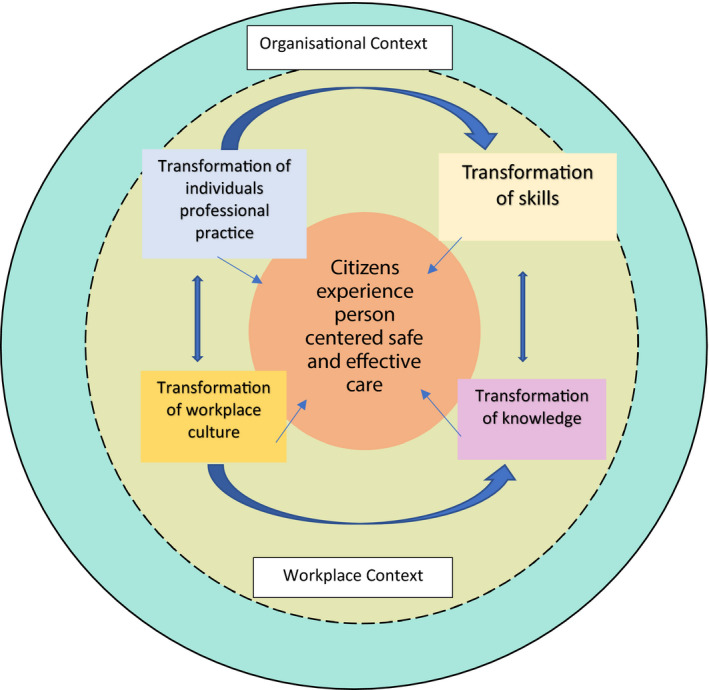
Model illustrating key purpose, context and components of contemporary CPD (Jackson et al., 2015)

“… *in order for CPD to be effective it has to address all of the outcomes for individual, team, service and organisational transformation because they are interrelated and interdependent*.” (Jackson et al., p.104). Therefore, it is important that the purpose of CPD is not only to transform an individual's practice, but also requires transformation of workplace culture and context, to achieve optimal impact for service users (citizens and professionals) (Figure 1).

Although there is a dearth of evidence on the effectiveness of CPD, there have been significant advances in defining potential indicators of impact. Indicators are defined as measurement tools used to monitor and evaluate the quality of important governance, management, clinical and support functions (JCAHO, 1990). Indicators found to be most helpful at different levels are identified in Table 2 mapped to the four transformation theories (Jackson et al., 2015).

**TABLE 2:** The outcomes and associated indicators most useful for measuring individual, team, service and organizational impact of CPD (Jackson et al., 2015)

Individual professional practice	Skills to meet service provision for society’s needs	Knowledge/knowledge translation	Workplace teams/context to deliver on organizational/system values
Role Clarity Skilled and competent Role Model Self‐awareness and confidence Emotional intelligence Compassion Person centred Speaking up for human rights Positive impact on patient experience Active Lifelong learning Critical reflection Career progression and personal growth Using evidence systematically Creative problem solving Positive attitude to change	Shared purpose Shared values Inclusive culture Whole systems working Systems for shared governance Organizational awareness and intelligence Good partner relations Commitment to lifelong learning Quality metrics Effective use of Resources Compliance with national standards Creativity and innovation PPI and public trust	Shared vision and purpose for service Integrated working Person‐centred culture Patient at heart of decision‐making Effective levels of staffing Patient experience and safety metrics Improved patient flow and discharge Systematic mechanism for capturing best and poor practice Reviewing and improving standards/Clinical Audit	Role clarity and responsibility Shared vision and values Interdisciplinary team working Person‐centred team culture Collaborative decision‐making Effective team communication Positive learning culture Commitment to lifelong learning Skilled facilitation of others High challenge and support Peer learning and review Innovation and creativity Systematic use of evidence to inform practice

Identifying what enables processes of learning and development helps us to understand the possible mechanisms through which CPD learning is achieved. Transformation occurs initially when CPD enables individual practitioners to become more self‐aware of the way in which their values, beliefs and attitudes influence their behaviour in the workplace. Developing role clarity and emotional intelligence about the way in which practitioners influence both workplace culture and delivery of person‐centred, safe and effective care, should be the primary goal of CPD programmes of learning. Psychological safety in the workplace is essential to enable practitioners to develop insight, confidence (Landor, 2011) and feel as though their contributions to the delivery of person‐centred, safe and effective care are valued.

Carpenter (2011) suggests that outcomes for service users and carers can generally be considered in terms of changes in such factors as the quality of life, skills and behaviour, self‐esteem and levels of stress (Carpenter, 2011). The RCN’s Principles of Nursing Practice (2010) detail what society (including colleagues, patients, or the families or carers of patients) should expect from nursing.

The evidence presented suggests the purpose of CPD should be refocused to emphasize learning in and from the workplace for direct benefit to service users and citizens, through transforming:

1. individuals to self‐directing, resilient self‐sufficient lifelong learners that drive their own learning;
2. the way different staff groups work together to become effective teams recognized by flexibility and team competences;
3. cultures so that workplaces live shared values, everyone flourishes, and knowledge is used in practice, to enable safe and effective practice to be experienced by service users in a person‐centred way.

## 4 CPD ISSUES REQUIRING ACTION

The NHS People Plan (2020) and NHS Long Term Plan (2019) have ambitions to transform the future delivery of health and social care through integrated “place‐based” care systems (ICSs) driven by citizen and population health needs. The plan requires the current and future multi‐professional workforce to have the capacity and capability to facilitate and lead integrated approaches to system‐wide transformation and be equipped to deliver quality services that are person‐centred, safe, effective and seamless, providing value for money, in increasingly complex circumstances (NHS Improvement, 2016).

Co‐ordinated and focussed CPD is an untapped resource for contributing to this strategy, but issues influencing its potential impact need to be resolved. From our extensive review of the evidence there are four key issues for nursing CPD requiring action:

1. how to strengthen the focus on patient experience as the starting point for CPD;
2. the lack of evidence of CPD effectiveness and accountability in its transfer to practice;
3. evaluation of CPD effectiveness; and
4. involving citizens in targeting CPD where it is most needed.

The paper presents each of these in turn below.

### 4.1 Issue 1: Patient experience is not used as a starting point for commissioning CPD

The assessment, measurement and evaluation of learning outcomes from CPD are underdeveloped and inconsistent (Academy of Medical Royal Colleges, 2010; Clark et al., 2008; Fryer, 2006; Illing et al., 2018; Jackson et al., 2015; Manley et al., 2018; Mathers, 2012) and, in spite of regulatory commitment to CPD, there is a lack of substantial evidence base to demonstrate the link between patient and citizen experiences of care, service improvements and practitioner behaviour change. This undermines confidence in the value of CPD for transforming the future workforce to meet citizen health needs (The Health Foundation, 2013).

We do not know what types of CPD are more effective and provide value for money in terms of return on investment. This is compounded by the fact that patient experience and outcomes are not the starting point for commissioning the CPD required to transform the workforce for driving improvements in care and patient safety, despite readily available data such as Friends and Family Tests and patient satisfaction surveys that could provide baseline measures to track CPD impact over time. Double‐loop feedback mechanisms built into commissioning processes would enable citizen experience of using health and social care services to drive allocation of CPD budgets and investment in the development of CPD metrics to demonstrate sustained change at all system levels.

Nursing CPD should not take place in a vacuum but within a multi‐professional frame focused on growing and retaining the workforce to enable all levels of practitioner to fulfil career aspirations. Health Education England has recognized this through the creation of multi‐professional advanced and consultant practitioner career frameworks (HEE, 2017; 2020), with the NHS Long Term plan (National Health Service, 2019). These focus on the provision of integrated health and social care which involve all professions and new roles to meet the future needs of populations across communities. However, it is not yet clear how these career frameworks will drive commissioning of CPD because the workforce modelling approaches in England are outdated and based on techno‐rationalistic models of CPD education. The focus for commissioners and regulators should therefore be on testing existing frameworks that measure impact and on working more closely with citizens to include their representation in planning future workforce transformation to build the capacity and capability of the workforce.

### 4.2 Issue 2: There is insufficient evidence of CPD effectiveness and lack of accountability in seeing through its transfer to practice or focusing on contextual factors

The ability to mobilize and combine the experiential knowledge of practitioners, citizens and service users, with the formal evidence from research is missing in existing CPD, especially when contextual factors namely; leadership, culture and an evaluation focus are pivotal to its implementation (Rycroft‐Malone et al., 2012). Cultural transformation requires more than traditional approaches to CPD because this is more fundamental to how people experience their work as highlighted during the COVID‐19 pandemic (Jackson et al., 2020). Nationally funded projects by HEE (Illing et al., 2018; Jackson et al., 2015; Manley et al., 2018), informed by public consultation have demonstrated how CPD can make a difference to the workplace, patient and staff outcomes and career progression if indicators of impact at all levels of the system are developed to inform a more coherent commissioning strategy.

Currently there is a lack of government funding for future workforce CPD and an increasing disconnect between developing the future workforce fast enough to meet the skills and staffing number deficits, whilst building capacity and capability for integrated working and systems transformation to deliver future services. There is a need to invest in workforce development programmes that provide flexible learning opportunities across systems using the workplace as the main resource for learning, development, improvement and innovation if the vision of the NHS Long Term Plan (2019) and NHS People Plan (2020) is to be achieved. The development of CPD process measures that demonstrate impact at all levels of the health and care system will help commissioners identify programmes that provide the most holistic impact for the workforce and citizens whilst delivering value for investment.

Without greater consideration of the effectiveness and cost‐effectiveness of the different forms of CPD, piecemeal approaches to CPD will persist (Moriarty, 2014). Post‐COVID‐19, greater effectiveness in using minimal resources and ever‐shrinking professional development budgets will be needed. Future CPD therefore must optimize opportunities to learn, develop and improve in through and from work because nurses may not be afforded time away from the workplace to think and reflect on their development – this requires skilled and holistic facilitation. More investment is needed in developing the facilitation skills and capacity of learning, development, quality and transformation practitioners working at the meso‐service systems level to enable front‐line teams to feel supported and empowered to contribute creatively to the solutions required. Additionally, the ability to facilitate the complex change needed for systems transformation also requires investment in the development of systems leaders with the integrated skill sets required to break down silos and boundaries for integrated ways of working and learning across the system (Manley et al., 2016).

Inter‐professional learning in, through, and from practice, collective leadership, and CPD is pivotal to the delivery and evaluation of sustainable transformation across the health economy to achieve future new models of care with a foundation in person‐centred values, relationships and shared decision‐making. Large‐scale transformation that draws out and mobilizes the talents and natural creativity of the workforce bottom‐up, underpins improvement in processes and outcomes. This is linked to creating positive conditions for change through work environments that harness relationships, skills and capabilities of individuals in the system, in contrast to many top‐down approaches that focus on control of change (Best et al., 2012; Lanham et al., 2009), thus reinforcing the importance of the microsystems level (front‐line teams and individuals) (Nelson et al., 2002). Focusing on how people work in large‐scale change is more important than attaining pre‐determined targets when working towards transformation (Best et al., 2012) and shared collective leadership is more effective than a hierarchical approach, as it gives staff autonomy in their work along with developing shared responsibility (West et al., 2017).

The Venus model for workforce transformation (Manley & Jackson, 2020), has the potential to fill the void of traditional CPD programmes, because it focuses on contextual and process determinants that enable transformation identifying 5 interdependent skill sets central to sustainable transformation: facilitation of integrated learning, development, improvement, knowledge translation, inquiry and innovation; team and systems leadership; practice development; improvement; and culture change.

Organizational contexts and processes through their cultures and leadership approaches influence their readiness to optimize workforce programmes for transformation and outcomes, and need to be assessed for readiness and customization, rarely attended to in the published evaluations of leadership programmes in the United Kingdom. Other authors have highlighted the importance of learning as a foundation for transformation (Crowe & Manley, 2019; Dixon‐Woods, 2019; Rycroft‐Malone et al., 2012).

What is needed is a holistic (Dixon‐Woods, 2019; Manley et al., 2019) and “bottom‐up” approach (drawing upon the experience and insights of those delivering services) for wider system learning (Cardiff et al., 2020; West et al., 2017, 2018).

It is vital therefore that future CPD models focus on developing the capacity and the capability of nursing as part of the wider multi‐professional workforce to develop an integrated approach to transformation which focuses on:

1. clinical/care systems leadership with all the skills required to support systems transformation at the macro‐level across partner boundaries towards a common purpose for service provision, multi‐professional workforce effectiveness and ongoing cultures of innovation based on shared governance and system‐wide learning for quality and staff outcomes;
2. team leadership at the micro‐level to support the development of effective workplace cultures associated with high performing teams and improved quality and staff outcomes;
3. facilitating an integrated approach, at the meso‐ and micro‐level that draws on all the skills required for supporting and enabling successful multi‐professional learning, development improvement and knowledge translation.

### 4.3 Issue 3: How to demonstrate impact of CPD and the requirement for metrics

When demonstrating CPD impact, it is important to measure what we value as a profession cognizant with the purposes of CPD, rather than valuing measurement per se. This means focusing less on measuring everything and more on what is most important, for example the impact on person‐centred care, safety, effectiveness, continuity and the contextual factors positively influencing these outcomes. The literature uses a confusing array of terms relevant to impact measurement, some are described in Table 3.

**TABLE 3:** Description of indicators, impact and outcomes (Jackson et al., 2015)

Metric descriptor	Definition
Indicator	Quantitative and qualitative evidence of the degree to which a result is occurring over time. They should be relevant; repeatable, verifiable and time‐bound.
Impact (Educational)	Is the demonstrable contribution that education makes to the economy, society, culture, public policy or services, health, the environment or quality of life, beyond contributions to academia. Assesses whether an intervention works in relation to its defined objectives.
Outcomes	The changes to people resulting from the activity, and measure progress towards achieving that change through an organization’s and/or systems work.

The CPD Impact Tool developed by Jackson et al., (2015), co‐created with a wide range of stakeholders, identifies indicators of CPD effectiveness at all system levels (Table 2) and has the potential for development into a set of CPD metrics for commissioners, providers and government as a national benchmarking tool. Additionally, meso‐level education practitioners whose role is to facilitate learning, development and improvement in the workplace could use them as a form of self‐assessment, and nurses could demonstrate achievements, gaps and challenges in CPD learning through educational passports for revalidation purposes.

In the United Kingdom, we need a standardized set of indicators to measure the impact of CPD and an educational approach for all professions underpinned by sound educational theory that recognizes the realities of practice. The four transformation theories highlighted in this paper have the potential to form the bedrock of CPD in the future.

### 4.4 Issue 4: How to work with communities to ensure CPD focuses on what matters to patients, citizens and communities?

Finally, there is a need to focus on what matters to citizens and service users of health care so that citizens feel more empowered to make their own choices and take responsibility for their own health. This shifts the lens from the paternalistic view of healthcare systems to a strength‐based model. The question then becomes how do we enable authentic engagement with citizens to achieve this? One solution may be for Higher Education Institutes (HEIs) providing CPD to transform the way they work when designing, delivering and evaluating programmes through stronger authentic engagement with and representation of citizens. There is scope for the UK Council of Deans for Health to develop a more coherent national strategy for citizen engagement and involvement in CPD. Huge potential exists to work with the private voluntary and independent sectors, local charities, local governments and citizens assemblies as partners in CPD to ensure that it can flex and adapt to the changing needs of populations and communities. Future investment, research and an evidence base for impact will identify how this is authentically achieved.

## 5 Summary

In summary, we propose that CPD is commissioned to meet the needs of patients, citizens and populations as the starting point, taking a strength‐based approach to co‐creating workforce development programmes that develop the capacity and capability of the workforce as a whole to deliver person‐centred, safe and effective evidence‐informed care. This involves shifting the lens from individuals and organizations to teams and systems built around facilitating the effective allocation of scant CPD resources to optimize benefit for patients, citizens and practitioners. CPD investment needs to focus on building the capacity of systems leaders, clinical team leaders and facilitators of learning, development and improvement using the workforce as the main resource and developing cultures of learning that foster psychological and holistic safety.

At a national level, it is vital that CPD is funded adequately by the UK government. However, CPD for the nursing profession cannot be in a vacuum, it must be linked to using the workplace as the main resource for active learning, development, improvement and innovation. With the development of greater focus on multi‐professional integrated care to meet the health needs of people there is also a need for greater fluidity across professions. This requires appropriate CPD accreditation models to be developed that reflect the importance of supporting nurses to learn at work. Embedded active learning with other professionals in the healthcare team in the workplace creates a learning culture in, through and from practice, which places the patient and the public, at the heart of learning so that improvements are generated to enhance patient and family experiences and person‐centred outcomes in real time.

## 6 CITIZEN ENGAGEMENT METHODS

Citizen Consensus panels were recruited by the University of Sheffield for consultation on a number of pre‐determined themes related to nursing regulation, workload, CPD and models of care. Each team of academic leaders in these fields were invited to write a contemporary evidence review paper which was shared with citizen panel members. Citizen panel members reviewed this evidence and invited each team of experts to present core themes of interest to them in a virtual conference, supported by a paper presenting these themes. Each team were given 20 min to present and then a further 40 min to debate the issues identified and address questions by the citizen panel members. Key themes were summarized in a report for the project leads. The Consensus methodology will be presented in a separate publication in the spring but is adapted from both a Scandinavian and Canadian context (Fassbender, 2018; Grundhal, 1995). The remainder of this section focuses on the feedback and key issues identified by citizens for nursing CPD.

## 7 CITIZEN FEEDBACK

The panel identified that nursing CPD is a complex topic with technical terms linked to nurse education and competencies. The panel raised questions about CPD content, motivations for organizations and individuals, resourcing and impact. There were 7 main issues discussed in by the Consensus panel and a summary provided below.

### 7.1 CPD is important for nurses, patients and organizations

The panel discussion highlighted that CPD was not just about skills training, it is also about lifelong learning that supports personal development and staff retention. The COVID‐19 pandemic demonstrates that nurses need transferable skills as they move between specialisms to support patients. CPD should not be seen in a narrow functional way, for example training sessions in a specific technique. The value in bringing nurses together in external CPD sessions was suggested as a means to aid mutual learning and help refresh thinking.

### 7.2 What sort of CPD is needed?

The evidence suggests that there was no “one size fits all” model for CPD and that a combination of methods is probably required. Investment in the right places is needed, but the panel heard that no one currently maps out CPD requirements for the workforce based on service user feedback. Currently, there is a fragmentation between university education, what health organizations need, and what policy makers think should be given for CPD. The panel highlighted that, increasingly, care is given in an integrated system, with the growing links between hospital and community and between health and social care. Nurses work across this integrated system and CPD should reflect this.

### 7.3 The link between CPD and retention of nurses

When nurses move from one organization to another or even leave the NHS to work in another healthcare role this could be considered a “loss to the system”, but any CPD they have undertaken continues to shape their practice. One suggestion was that CPD could be considered as an investment in the human capital (i.e. the staff) of the NHS and that in turn promotes better patient outcomes across the whole health system. There was some discussion on the merits of tying high quality CPD to qualifications and promotion, with a stipulation that funding should come with a requirement to stay in an organization for a period.

### 7.4 Measuring how effective CPD is

Questions were raised about the measurement of the impact of CPD on patient outcomes and person‐centred care. The discussion emphasized the need to consider the impact of resource allocation on patient experiences and ensure that patient and citizen experiences are included in designing learning for CPD. There was a feeling that surveys such as Friends and Family Tests and patient satisfaction surveys were meaningless if they are not used as a starting point for CPD commissioning and design to address gaps in quality of care and services provided.

### 7.5 Ensuring equity of access to CPD

There were questions about the adequacy of funding for CPD and how this affected learning opportunities. There was some concern expressed, linked to personal experiences, that the aspirations and policy statements on CPD do not trickle down to the nurses who are working on wards and in community settings. The panel agreed that self‐funding and reliance on learning on the job did not lead to best nursing practice. The importance of CPD being linked to appraisal and nurses seeing CPD as something that went beyond the mandatory minimum was discussed. A question was asked about whether sponsorship by private companies can adversely affect what CPD is available and introduce bias.

### 7.6 The need for a supportive culture

The panel reflected on how responsibility for funding CPD should not just rest with the individual nurse. Healthcare organisations have a role to play and a culture change is needed to support CPD and time to study. The evidence suggests that culture and leadership is important for attracting and retaining nurses. CPD learning will be applied where there is a positive culture and leadership around person‐centred care.

### 7.7 More public involvement in CPD strategy

There was some discussion centred on the role of Patient and Public Involvement (PPI) in CPD, as community involvement was raised as a contested issue in the presentation. There was scope for having PPI panels focused on CPD, better clinical‐academic links and ensuring the public are involved in decisions regarding CPD funding. This could mirror the way PPI is a major part of research funding strategy quite easily. Overall, the panel considered that there is an opportunity for citizens to play a greater role in driving the CPD agenda.

In summary, the Consensus methodology used in this study was extremely beneficial to both the academic research team and the citizens. The approach focused attention on relaying key messages that resonated with the experts by experience in a format that was more readily digestible through presentation and key focused supportive papers. It enabled a critical free‐flowing dialogue that provided real‐time feedback on what could be celebrated from current understanding, Consensus over challenges and what actions need to be taken. Our recommendation would be to build this into further work to develop CPD if it is to authentically engage members of the public in a co‐design model to benefit local communities.

## 8 CONCLUSION

Our research highlights that a holistic approach to workforce development is required at all system levels for quality care and transformation of services involving all interdependent partners, working with the complexity of practice contexts, using the workplace as the main resource for learning, development and improvement (Manley & Jackson, 2020; Manley et al., 2016, 2019; Martin & Manley, 2018). Changing the model of funding, supporting and embedding CPD that uses the workplace in this way has the potential to create a future workforce who strive to continually grow knowledge and skills throughout their career, within their context, with their colleagues and in partnership with citizens.

It is important to recognize that our current understanding of how and where CPD should be best provided and the skill sets required to facilitate learning in the workplace, needs to be challenged (Manley & Jackson, 2020). Workplace culture plays an important part in enabling knowledge transfer to embed learning and informs organizational and systems learning (Manley & Jackson, 2020). Future attention should be focused on how we create psychological and holistic safety in enabling learning cultures across all system levels. This can only be achieved by investing in the future development of systems leaders who have the skill set to facilitate integration at a system‐wide level. Having clear system architecture, resources, time and support to nurture this is essential, and it is hoped that the future NHS Spending Reviews and NHS Bill will recognize the importance of investing in the right parts of the system to enable this to happen.

There is little evidence of the impact of CPD in providing safe and effective patient care (King et al., 2021) and it is essential to recognize the importance of taking a whole systems approach to this end for the future. To date, researchers have developed frameworks that have the potential to measure CPD impact for citizens and at the individual, team, organization and system level and further work is taking place to further test and evaluate these (Illing et al., 2018; Jackson et al., 2015; Manley et al., 2018).

This offers a significant challenge to policy makers, commissioners, regulators, HEIs and providers to work together to plan for multi‐professional workforce transformation focusing on development of an integrated skill set for innovation and improvement, underpinned by research of evidence of impact, starting with patient and citizen experience as the driving force for commissioning.

**Take home messages:**

1. In rapidly changing healthcare contexts, it is vital to understand the synergistic relationship between individual aspiration, workplace context and culture, if CPD opportunities are to be maximized to promote high quality, person‐centred care.
2. Developing individuals as whole system leaders can promote team effectiveness and help transform workplace culture in ways that increase patient benefit.
3. Future studies should seek to measure the value of CPD for citizens and communities experiencing care, test the feasibility of new CPD impact tools and metrics and gain evidence of cost‐effectiveness of CPD at all levels of the system for return on investment. (King et al., 2021; Manley & Jackson, 2020).
4. A clearer mechanism for commissioning CPD needs to be developed to enable funders and regulators to understand the impact of nursing CPD on the quality and safety of services and citizen experience.
5. CPD hours should be matched to European standards and investment made to enable nurses to achieve regulatory requirements for CPD, through time, financial resources and a supportive workplace culture at all levels of the system.

## CONFLICT OF INTEREST

There are no known conflicts of interest associated with this work.

## ETHICAL APPROVAL

Ethical approval was not needed for this piece of work as we were commissioned by the RCN Strategic Research Alliance so for the fuller piece any ethics lies with the lead partner University of Sheffield. The original study in 2015 was covered by University ethics.

## Data Availability

All data from the original studies cited by Manley and Jackson and Jackson et al in this paper are available on request.

## ENDNOTES

^1^
We use the term system to describe all the interdependent partners working together to provide integrated health and social care services to meet the needs of citizens, communities and local populations.

^2^
Level of Staff Burnout Real Concern for NHS Leaders. Nursing Times, 6 October 2020, https://www.nursingtimes.net/news/workforce/level‐of‐staff‐burnout‐real‐concern‐for‐nhs‐trust‐leaders‐0610‐2020/

## References

[nop2941-bib-0001] Academy of Medical Royal Colleges . (2010). The Effectiveness of Continuing Professional Development Final Report. College of Emergency Medicine. [Online]. The Academy of Medical Royal Colleges. Available at: http://www.aomrc.org.uk/doc_view/213‐effectiveness‐of‐cpd‐final‐report

[nop2941-bib-0002] Aiken, L. H. , Sloane, D. M. , Ball, J. , Bryneel, L. , Rafferty, A. M. , & Griffiths, P. (2018). Patient satisfaction with hospital care and nurses in England: An observational patient study. British Medical Journal Open, 8(1), e019189. 10.1136/bmjopen-2017-019189 PMC5781188 29326193

[nop2941-bib-0003] Best, A. , Greenhalgh, T. , Lewis, S. , Saul, J. E. , Carroll, S. , & Bitz, J. (2012). Large‐system transformation in health care: A realist review. Milbank Quarterly, 90(3), 421–456. 10.1111/j.14680009.2012.00670.x PMC3479379 22985277

[nop2941-bib-0004] Cardiff, S. , Sanders, K. , Webster, J. , & Manley, K. (2020). Guiding lights for effective workplace cultures that are also good places to work. International Practice Development Journal, November. 10(3), 1–20. https://www.fons.org/library/journal/volume10‐issue2/article2v2

[nop2941-bib-0005] Carpenter, J. (2011). Evaluating social work education: A review of outcomes, measures, research designs and practicalities. Social Work Education, 30(2), 122–140. 10.1080/02615479.2011.540375

[nop2941-bib-0006] Chaudhry, F. B. , & Raza, S. (2020). COVID 19: Frontline experience at a tertiary care hospital in UK. Journal of Global Health, 10(1), 1–5. 10.7189/jogh.10.010356 PMC7242887 32509287

[nop2941-bib-0007] Clark, E. , Draper, J. , & Sparrow, S. (2008). The impact on practice (ImP) project: A project to develop a framework to evaluate the impact of continuing professional education on practice. In: 19th Annual International Nurse Education Tomorrow conference, 2–4 September 2008, Cambridge University.

[nop2941-bib-0008] Council of Deans of Health . (2016). A False Economy: Cuts to Continuing Professional Development funding for nursing, midwifery and the Allied Health Professions in England. Council of Deans of Health. https://councilofdeans.org.uk/wp‐content/uploads/2016/09/19092016‐A‐False‐Economy‐CPD‐cutsin‐England‐2016‐17‐.pdf

[nop2941-bib-0009] Coventry, T. (2015). Organizational impact of nurse supply and workload on nurses continuing professional development opportunities: An integrative review. Journal of Advanced Nursing, 71(12), 2715–2727. 26148213 10.1111/jan.12724

[nop2941-bib-0010] Crowe, C. , & Manley, K. (2019). Assessing contextual readiness: The first step towards maternity transformation. International Practice Development Journal, 9(2), Article 6 November. 1–20. 10.19043/ipdj.92.006

[nop2941-bib-0011] Dixon‐Woods, M. (2019). How to improve health care improvement – an essay by Mary DixonWoods. BMJ, 367, 15514. 10.1136/bmj.l5514 PMC6768008 31575526

[nop2941-bib-0013] European Union Health Programme (2013). Study concerning the review and mapping of continuous professional development and lifelong learning for health professionals in the EU. European Union.

[nop2941-bib-0014] Fassbender, K. (2018). Consensus development conference: Promoting access to quality palliative care in Canada. Journal of Palliative Medicine. 21(S1), S1‐S8.January. 10.1089/jpm.2017.0453 PMC5733647 29283875

[nop2941-bib-0015] Fryer, R. H. (2006). Learning for a change in healthcare. Department of Health and the NHS.

[nop2941-bib-0016] Graham, I. , Logan, J. , Harrison, M. B. , Straus, S. , Tetroe, J. , Caswell, W. , & Robinson, N. (2006). Lost in knowledge translation: Time for a map? Journal of Continuing Education for Health Professionals, 26(1), 13–24. 10.1002/chp.47 16557505

[nop2941-bib-0017] Greenberg, N. , Docherty, M. , Gnanapragasam, S. , & Wessely, S. (2020). Managing mental health challenges faced by healthcare workers during covid‐19 pandemic. BMJ, 368, m1211. 32217624 10.1136/bmj.m1211

[nop2941-bib-0018] Grundhal, J. (1995). The Danish Consensus conference model. Public participation in science: The role of Consensus conferences in Europe 1995 (pp. 31–40). Science Museum.

[nop2941-bib-0019] Health Education England (2017). Advanced Practice Frameworks. Health Education England. https://www.hee.nhs.uk/ourwork/advanced‐clinical‐practice/multi‐professional‐framework

[nop2941-bib-0020] Health Education England (2020). Multiprofessional Consultant Practitioner Framework. Health Education England. https://www.hee.nhs.uk/our‐work/advanced‐clinical‐practice/multi‐professional‐framework

[nop2941-bib-0021] House of Commons Health Committee (2018). The nursing workforce: Second report of session 2017–2019. House of Commons.

[nop2941-bib-0022] Illing, J. , Corbett, S. , Kehoe, A. , Carter, M. , Hesselgreaves, H. , Crampton, P. , & Ikah, D. (2018). How Does the Education and Training of Health and Social Care Staff Transfer to Practice and Benefit Patients? A Realist Approach. Newcastle University: Durham University: University of York. University of Newcastle.

[nop2941-bib-0023] Jackson, C. , Manley, K. , Webster, J. , & Hardy, S. (2020). Learning from first wave Covid‐19 across the system. Unpublished research report. University of East Anglia.

[nop2941-bib-0024] Jackson, C. , Manley, K. , Webster, J. , & Hardy, S. (2021). System wide learning from first wave Covid 19: A realist synthesis of what works? Research Square. 10.21203/rs.3.rs-115647/v1. In review

[nop2941-bib-0025] Jackson, C. , Manley, K. , Martin, A. , & Wright, T. (2015). Continuing professional development for quality care: Context, mechanisms, outcomes and impact. Centre for Practice Development, Canterbury Christ Church University. Final Report ECPD ISBN 978‐1‐909067‐39‐4.

[nop2941-bib-0026] Joint Commission on Accreditation of Healthcare Organizations (1990). Primer on indicator development and application: Measuring quality in health care. Oakbrook Terrace, Illinois: Joint Commission on Accreditation of Healthcare Organizations.

[nop2941-bib-0027] King, R. , Taylor, B. , Talpur, A. , Jackson, C. , Manley, K. , Ashby, N. , Tod, A. , Ryan, T. , Wood, E. , Senek, M. , & Robertson, S. (2021). Factors that optimise the impact of continuing professional development in nursing: A rapid evidence review. Nurse Education Today, 98, 104652. 10.1016/j.nedt.2020.104652 33190952

[nop2941-bib-0028] Kitson, A. , Harvey, G. , & McCormack, B. (1998). Enabling the implementation of evidence based practice: A conceptual framework. Quality in Health Care, 7(3), 149–158. 10185141 10.1136/qshc.7.3.149 PMC2483604

[nop2941-bib-0029] Landor, M. (2011). Is the glass half‐full or half‐empty? Perceptions of recently‐qualified educational psychologists on the effectiveness and impact of their Master’s level research. Educational Psychology in Practice, 27(1), 83–95. 10.1080/02667363.2011.549356

[nop2941-bib-0030] Lanham, H. J. , McDaniel, R. R. , Crabtree, B. F. , Miller, W. L. , Stange, K. C. , Tallia, A. F. , & Nutting, P. A. (2009). How improving practice relationships among clinicians and non‐clinicians can improve quality in primary care. Joint Commission Journal on Quality Patient Safety, 35(9), 457–466. 19769206 10.1016/s1553-7250(09)35064-3 PMC2928073

[nop2941-bib-0031] Maben, J. , Adams, M. , Peccei, R. , Murrells, T. , & Robert, G. (2012). Poppets and parcels: The links between staff experience of work and acutely ill older peoples’ experience of hospital care. International Journal of Older People Nursing, 7(2), 83–94. 22531048 10.1111/j.1748-3743.2012.00326.x

[nop2941-bib-0032] Maben, J. , & Bridges, J. (2020). Covid‐19: Supporting nurses' psychological and mental health. Journal of Clinical Nursing, 29, 2742–2750. 10.1111/jocn.15307 32320509 PMC7264545

[nop2941-bib-0033] Manley, K. , & Jackson, C. (2020). The Venus model for integrating practitioner‐led workforce transformation and complex change across the health care system. Journal of Evaluation in Clinical Practice, 26(2), 622–634. 32172538 10.1111/jep.13377

[nop2941-bib-0034] Manley, K. , Jackson, C. , & McKenzie, C. (2019). Microsystems culture change: A refined theory for developing person‐centred, safe and effective workplaces based on strategies that embed a safety culture. IPDJ, 9(2), 1–21.

[nop2941-bib-0035] Manley, K. , Martin, A. , Jackson, C. , & Wright, T. (2016). Using systems thinking to identify workforce enablers for a whole systems approach to urgent and emergency care delivery: A multiple case study. BMC Health Services Research, 16, 368. 10.1186/s12913-016-1616-y 27507157 PMC4979146

[nop2941-bib-0036] Manley, K. , Martin, A. , Jackson, C. , & Wright, T. (2018). A realist synthesis of effective continuing professional development (CPD): A case study of healthcare practitioners' CPD. Nurse Education Today, 69, 134–141. 10.1016/j.nedt.2018.07.010 30059819 PMC6278905

[nop2941-bib-0038] Martin, A. , & Manley, K. (2018). Developing standards for an integrated approach to workplace facilitation for interprofessional teams in health and social care contexts: A Delphi study. Journal of Interprofessional Care, 32(1), 41–51. 10.1080/13561820.2017.1373080 29058564

[nop2941-bib-0039] Marvin, S. , Lee, L. , & Robson, F. (2010). The evaluation of learning and development in the workplace: A review of literature. Higher Education Funding Council for England.

[nop2941-bib-0040] Mathers, N. , Mitchell, C. , & Hunn, A. (2012). A study to assess the impact of continuing professional development (CPD) on doctors’ performance and patient/service outcomes for the GMC. Capita Business Services. 13. University of Sheffield, Capita Health Ltd..

[nop2941-bib-0041] Moriarty, J. (2014). Post‐qualifying education for social workers: A continuing problem or a new opportunity? Social Work Education, 33(3), 397–411.

[nop2941-bib-0042] National Health Service (2019). The NHS Long term Plan. NHS England. https://www.longtermplan.nhs.uk/publication/nhs‐long‐term‐plan/

[nop2941-bib-0044] Nelson, E. C. , Batalden, P. B. , Huber, T. P. , Mohr, J. J. , Godfrey, M. M. , Headrick, L. A. , & Wasson, J. H. (2002). Microsystems in health care: Part 1. Learning from high‐performing front‐line clinical units. Joint Commission Journal on Quality Improvement, 28(9), 472–493. 10.1016/s1070-3241(02)28051-7 12216343

[nop2941-bib-0045] NHS Improvement (2016). Developing People, Improving Care. NHS Improvement. https://improvement.nhs.uk/resources/developing‐people‐improving‐care

[nop2941-bib-0046] Nursing and Midwifery Council (2017). How to revalidate with the NMC. NMC. https://www.nmc.org.uk/globalassets/sitedocuments/revalidation/how‐to‐revalidate‐booklet.pdf

[nop2941-bib-0047] Nursing and Midwifery Council (2018). Future Nurse: Standards for Proficiency for Registered Nurses. Nursing and Midwifery Council. Available from: https://www.nmc.org.uk/globalassets/sitedocuments/educationstandards/futurenurse‐proficiencies.pdf 10.1016/j.nedt.2024.106284 38870582

[nop2941-bib-0048] Rhydderch, M. , Elwyn, G. , Marshall, M. , & Grol, R. (2004). Organisational change theory and the use of indicators in general practice. Quality and Safety in Health Care, 13(3), 213–217.PMID: 15175493; PMCID: PMC1743845. 10.1136/qhc.13.3.213 15175493 PMC1743845

[nop2941-bib-0049] Rowley, E. , Morriss, R. , Currie, G. , & Schneider, J. (2012). Research in practice: collaboration for Leadership in Applied Health Research and Care (CLAHRC) for Nottinghamshire, Derbyshire, Lincolnshire (NDL). Implementation Science, 3(7), 40. 10.1186/1748-5908-7-40 PMC3441357 22553966

[nop2941-bib-0051] Royal College of Nursing (2016). RCN factsheet: Continuing Professional Development (CPD) for nurses working in the United Kingdom (UK). RCN.

[nop2941-bib-0052] Royal College of Nursing (2018). Investing in a safe and effective workforce: Continuing professional development for nurses in the UK. RCN.

[nop2941-bib-0053] Rycroft‐Malone, J. O. , McCormack, B. , Hutchinson, A. M. , DeCorby, K. , Bucknall, T. K. , Kent, B. , Schultz, A. , Snelgrove‐Clarke, E. , Stetler, C. B. , Titler, M. , Wallin, L. , & Wilson, V. (2012). Realist synthesis: Illustrating the method for implementation research. Implementation Science, 7(33), 1–10. 10.1186/1748-5908-7-33 PMC3514310 22515663

[nop2941-bib-0054] The Health Foundation (2013). Measuring patient experience. The Health Foundation. https://www.health.org.uk/publications/measuring‐patient‐experience

[nop2941-bib-0055] Tran, D. , Tofade, T. , Thakkar, N. , & Rouse, M. (2014). US and international health professions' requirements for continuing professional development. American Journal of Pharmaceutical Education, 78(6), 129. 10.5688/ajpe786129 25147401 PMC4140495

[nop2941-bib-0056] Tynjälä, P. (2013). Toward a 3‐P model of workplace learning: a literature review. Vocational Learning, 6, 11–36.

[nop2941-bib-0057] West, M. (2018). It’s not about the money, staff engagement comes first. London: The Kings Fund. https://www.kingsfund.org.uk/blog/2018/03/staff‐engagement‐comes‐first

[nop2941-bib-0058] West, M. , Eckert, R. , Collins, B. , & Chowla, R. (2017). Caring to change: How compassionate leadership can stimulate innovation in health care. London: The Kings Fund.

